# Health System Resilience in the Eastern Mediterranean Region: Perspective on the Recent Lessons Learned

**DOI:** 10.2196/41144

**Published:** 2022-12-21

**Authors:** Mirwais Amiri, Mohannad Al Nsour, Alvaro Alonso-Garbayo, Abdulwahed Al Serouri, Adna Maiteh, Elsheikh Badr

**Affiliations:** 1 Global Health Development Eastern Mediterranean Public Health Network Amman Jordan; 2 World Health Organization Amman Jordan; 3 Ministry of Public Health and Population San'a Yemen; 4 Sudan Medical Specialization Board Khartoum Sudan

**Keywords:** health systems resilience, resilience, vulnerability, public health, Eastern Mediterranean Region countries, COVID-19

## Abstract

**Background:**

Public health has a pivotal role in strengthening resilience at individual, community, and system levels as well as building healthy communities. During crises, resilient health systems can effectively adapt in response to evolving situations and reduce vulnerability across and beyond the systems. To engage national, regional, and international public health entities and experts in a discussion of challenges hindering achievement of health system resilience (HSR) in the Eastern Mediterranean Region, the Eastern Mediterranean Public Health Network (EMPHNET) held its seventh regional conference in Amman, Jordan, between November 15 and 18, 2021, under the theme “Towards Resilient Health Systems in the Eastern Mediterranean: Breaking Barriers.” This viewpoint paper portrays the roundtable discussion of experts on the core themes of that conference.

**Objective:**

Our aim was to provide insights on lessons learned from the past and explore new opportunities to attain more resilient health systems to break current barriers.

**Methods:**

The roundtable brought together a panel of public health experts representing Field Epidemiology Training Programs (FETPs), Centers for Disease Control and Prevention in Atlanta, World Health Organization, EMPHNET, universities or academia, and research institutions at regional and global levels. To set the ground, the session began with four 10-12–minute presentations introducing the concept of HSR and its link to workforce development with an overall reflection on the matter and lessons learned through collective experiences. The presentations were followed by an open question and answer session to allow for an interactive debate among panel members and the roundtable audience.

**Results:**

The panel discussed challenges faced by health systems and lessons learned in times of the new public health threats to move toward more resilient health systems, overcome current barriers, and explore new opportunities to enhance the HSR. They presented field experiences in building resilient health systems and the role of FETPs with an example from Yemen FETP. Furthermore, they debated the lessons learned from COVID-19 response and how it can reshape our thinking and strategies for approaching HSR. Finally, the panel discussed how health systems can effectively adapt and prosper in the face of challenges and barriers to recover from extreme disruptions while maintaining the core functions of the health systems.

**Conclusions:**

Considering the current situation in the region, there is a need to strengthen both pandemic preparedness and health systems, through investing in essential public health functions including those required for all-hazards emergency risk management. Institutionalized mechanisms for whole-of-society engagement, strengthening primary health care approaches for health security and universal health coverage, as well as promoting enabling environments for research, innovation, and learning should be ensured. Investing in building epidemiological capacity through continuous support to FETPs to work toward strengthening surveillance systems and participating in regional and global efforts in early response to outbreaks is crucial.

## Introduction

Public health has a pivotal role in strengthening resilience at individual, community, and system levels as well as building resilient and healthy communities. During crises, resilient health systems can effectively adapt in response to evolving situations and reduce vulnerability across and beyond the systems. Health system resilience (HSR) is defined as follows [1,2]:

[T]he ability to prepare for, manage (absorb, adapt, and transform) and learn from shocks.

Hence, it is a key factor to coping with a crisis such as the economic crisis and the COVID-19 pandemic [2]. Although catastrophe risk reduction is based on the concept of resilience, it is only recently been applied to health systems. It has been widely characterized as institutions and health actors’ capacities to prepare for, recover from, and absorb shocks while sustaining basic activities and fulfilling the community’s continuing and acute care needs [3,4].

Even though the 680 million people of Eastern Mediterranean Region (EMR) make up only 9% of the global population [5], it is home to 43% of those in need of humanitarian assistance [6] and is the source of 64% of the world’s refugees [7]. The current resilience of health systems in the EMR varies from country to country, mostly based on the governmental and financial situation of the countries [8].

However, for the achievement of HSR, individuals, communities, and systems must be enabled to address challenges, such as poverty, inequality, unemployment, and other factors that endanger health and well-being. Relative success for HSR will depend on how the existing health systems can benefit from the newly learned lessons [9].

Global Health Development (GHD) is a regional initiative created to support countries in the EMR and strengthen their health systems to respond to public health challenges and threats. GHD was initiated to advance the work of the Eastern Mediterranean Public Health Network (EMPHNET) by building coordinating mechanisms with Ministries of Health, international organizations, and other institutions to improve population health outcomes. Serving as a collaborative platform, GHD|EMPHNET is dedicated to serving the region by supporting efforts to promote public health policies, strategic planning, sustainable financing, resource mobilization, public health programs, and other related services.

GHD|EMPHNET and the EMR’s Field Epidemiology Training Programs (FETPs) have made significant contributions to preparing for and responding to the present COVID-19 crisis. GHD|EMPHNET has the scientific competence to help raise country alert and preparation in the EMR and provide technical support through health promotion, training and training materials, guidelines, coordination, and communication. The FETPs are now involved in surveillance and screening at ports of entry, the development of communication materials and guidelines, as well as the dissemination of information to health professionals and the general public. However, several countries are still underequipped, have inadequate diagnostic capabilities, and require more capacity development to respond to public health issues. It is critical that GHD|EMPHNET and FETPs continue to create capacity to respond to COVID-19 and increase support for public health emergency preparedness and response [10]. If broadly and efficiently shared, there are crucial lessons that can help countries improve their preparedness, response, and strategy to tackle future health issues. Our local, national, regional, and global capacities face a variety of concerns and problems in this new era. Our region (ie, the EMR), like the rest of the world, must prepare for and respond to such threats by taking necessary measures. HSR, entailing the ability to adapt and thrive in the face of obstacles and hurdles, is more important than ever to recover from today’s extreme disruptions while preserving basic health system operations.

To engage national, regional, and international public health entities and experts in a discussion of public health challenges hindering the achievement of HSR in the EMR countries, EMPHNET held its seventh regional conference in Amman, Jordan, between November 15 and 18, 2021, under the theme “Towards Resilient Health Systems in Eastern Mediterranean: Breaking Barriers.” A panel of public health experts discussing the core theme of the conference represented FETPs, Centers for Disease Control and Prevention in Atlanta, World Health Organization, EMPHNET, universities or academia, and research institutions at regional and global levels. This viewpoint aims to portray the “Health System Resilience (HSR)” roundtable that took place during the conference.

## Objectives

The aim of our viewpoint is to provide insights on lessons learned from the past and explore new opportunities to attain more resilient health systems to break current barriers.

## Roundtable Panel Discussion

The roundtable brought together a panel of public health experts and to set the ground for the discussion, the session began with 10-12–minute presentations introducing the concept of HSR and its link to workforce development. The presentations were followed by an open question and answer session to allow for an interactive debate among panel members and the roundtable audience.

The panel of experts discussed challenges faced by health systems and lessons learned during the new public health threats to move toward more resilient health systems, overcome current barriers, and explore new opportunities to enhance the health systems’ resilience. Moreover, they presented the role of National Public Health Institutes in HSR and reviewed the role of the FETPs in strengthening the health systems’ resilience in the EMR.

The roundtable panel presented a structured approach to the resilience-building processes of health systems and formulated implementation of building back better approach; they also discussed the role of FETPs during the COVID-19 pandemic and other priority emergencies in the EMR. The panel discussion included oral presentations and an interactive discussion of questions and comments from participants, which covered the following topics: introduction to HSR—transforming challenges into opportunities, national public health institutes, and health systems’ resilience; the contribution of the health workforce to health system’s resilience—empirical and personal experiential examples; building resilient health systems—the role of the FETPs and Yemen FETP experience; and overall reflections about the presented topics and the lessons learned.

The panelist stressed that HSR is about transforming challenges into opportunities. An organized approach to resilience-building will ensure smooth recovery from emergencies and crises such as those observed during the COVID-19 pandemic or any other emergencies in the region and beyond. The panel members saw resilient health systems as a priority for all countries in the region. Adequate investments in health for socioeconomic development, investments for emergency preparedness, as well as an integrated approach to health security and universal health coverage are warranted. This should be supplemented by a robust primary health care foundation, investments in the essential public health functions, application of the whole-of-society approach, and attention to vulnerable and marginalized groups.

The ability to learn from country responses to emergencies like the last COVID-19 outbreak depends on the resilience of health systems. In this context, the panel confirmed earlier findings of an assessment of COVID-19 responses in 28 countries [11], which emphasized the activation of comprehensive response mechanisms, adapting health system capacity with cost-effective measures such as public-private partnerships, preserving health system functions and resources, and adopting effective measures to reduce vulnerability.

On the other hand, strong national public health institutes (NPHIs) [12] help countries avoid, detect, and respond to public health hazards more effectively. NPHIs are science-based organizations that lead and coordinate critical public health responsibilities. They are often housed in the government’s ministries of health or are closely associated with them. By generating, integrating, and interpreting public health data to make timely recommendations, NPHIs play a key role in health policy and decision-making during crises and challenging settings. NPHIs aid in the integration of public health decision-making activities by bringing together the required evidence and by coordinating efforts across sectors.

The contribution of the health workforce to health systems’ resilience was another area that was extensively discussed. The health workforce is considered the most important element for a health system to be able to absorb the increased health needs generated by shocks, adapt to deliver services with often less resources than usual, and transform themselves according to changes in the environment generated by the shock. However frontline health workers are often the most vulnerable individuals, such as in the case of epidemics, and often in armed conflicts when they become primary targets [1,13,14]. During the discussion, empirical examples were also given on the contribution of the health workforce to health systems’ resilience, for example, the flexible approach adopted by the government in Zimbabwe to adapt policies for deployment of health workers. This allowed for better retention, which in turn increased the capacity of the health system to continue delivering services in remote hard-to-reach areas even during the peak of the socioeconomic crisis [15]. Postconflict Timor-Leste, with a vast majority of population living in rural areas, is nowadays more able to address their public health needs after the contribution of Cuban government in producing more than 1000 medical professionals with a public health orientation. It is to be noted that this intervention greatly contributed to transform the health system’s focus from a “curative or hospital-centered” to a more “primary health care–based” system [16]. Within the EMR, the Lebanese health system adopted some workforce measures to be more able to cope with the increased health needs caused by the massive influx of Syrian refugees between 2012 and 2014. On the one hand, the Lebanese authorities increased nurses’ salaries to increase their attraction and retention, and on the other, they increased the number of scholarships available for applicants to nursing studies to increase production and availability of this key cadre [16]. According to Physicians for Human Rights reports of 2016, the Syrian health system decision makers decided to relax and adapt their regulations to allow physicians engaged in surgery training to undertake operations to address the increased workload generated by the conflict in Aleppo. Other examples of health workforce interventions that increased the resilience of health systems based on field experience include task-shifting (ie, allowing lower cadres to assume responsibilities previously attributed to higher level cadres). This was adopted in Angola during the armed conflict in 1975-2002 and in Pakistan after the earthquake of 2005. Other interventions reported include training of community health workers to assume basic public health functions. The example of the network of community health workers deployed in refugee camps during the massive influx into Tanzania following the genocide of 1994 in Rwanda was given. Finally, another example reported was the spontaneous initiative adopted by private providers in Syria to cover gaps in home care of patients with COVID-19 left by the public system in Syria in 2020-2021.

The panel also discussed how the FETPs are designed to build resilient public health systems and strengthen those systems through increasing the number and quality of field epidemiologists in the public health workforce. Such workforce is crucial for timely detection, investigation, and response to public health emergencies. By improving capacity to collect public health data through improved disease surveillance systems and using the collected data effectively, the application of evidence-based approaches in public health decision-making and policies can be promoted [17].

It has been shown that today more than 19,000 FETP alumni are trained worldwide, as the “boots on the ground” to detect and respond to public health threats, including infectious disease outbreaks, chronic and noncommunicable diseases, natural disasters, and humanitarian crises [18]. Having such workforce on the ground, responsive as quickly as possible to any emergency situation worldwide, would not have been possible without the investment in establishing FETPs [19].

The US Centers for Disease Control and Prevention and the World Health Organization have reinforced the central role of the FETPs for strengthening capacities of the health workforce in emergency management for greater resilience and health care response capacity [20].

The emergence and reemergence of infectious and noninfectious diseases is a major issue of public health concern. Previous outbreaks (eg, Ebola, Lassa fever, and cholera) have highlighted the need for a multisectoral public health workforce with the requisite competencies to effectively address these situations [21-23].

Today, the ongoing COVID-19 pandemic has highlighted the need for a well-trained public health workforce to save lives through timely outbreak detection and response [24,25].

Studies from different parts of the world have shown that since the early phase of COVID-19 epidemic, FETP trainees and graduates have been mobilized to support preparedness and response activities in their countries, and they have played a crucial role in HSR.

A survey of 65 FETPs around the world indicated that FETP residents and graduates have engaged in the COVID-19 response across all 6 regions of the World Health Organization. Response efforts focused on country-level coordination (98%), surveillance, rapid response teams, case investigations (97%), activities at points of entry (92%), as well as risk communication and community engagement (82%). Descriptions of FETP contributions to COVID-19 preparedness and response are categorized into the following 7 main themes: conducting epidemiological activities, managing logistics and coordination, leading risk communication efforts, providing guidance, supporting surveillance activities, training and developing the workforce, and holding leadership positions [26].

In the EMR, where the current COVID-19 situation continues to be of concern, the FETPs have also actively participated in surveillance and screening at the ports of entry, development of communication materials and guidelines, as well as sharing information with health professionals and the public [10]. Furthermore, The FETP graduates are found to be fully aware of the epidemiology of COVID-19 and the safety measures required, and they were well positioned to investigate and respond to the COVID-19 pandemic [27].

During the panel, the situation in Yemen, a country witnessing a protracted conflict limiting the capacity for public health and outbreak response, was discussed. Despite difficulties, the Yemen FETP has been instrumental in supporting COVID 19 response through participating in country-level coordination, planning, and monitoring, in addition to developing guidelines, standard operating procedures, and strengthening surveillance capacities. Other contributions of the program included outbreak investigations, contact tracing, case management, infection prevention and control, risk communication, and evidence-based research [28].

The panel members underlined that the COVID-19 pandemic and the varied national-level responses have reinforced the need for countries to invest in a trained public health workforce. The countries have learned that epidemiological skills are valuable assets and that other skills and disciplines such as data science need to be part of the modern field epidemiology training. Finally, the available evidence on the key role of the FETPs to support country response and ensure better HSR should reinforce the need for countries to invest in a trained public health workforce. The FETPs not only have the power to improve health systems during normal times but also can make health systems resilient to crises that may emerge over the longer run. This part of the panel discussion highlighted that a resilient health system can only be capable of managing the emerging and reemerging crises when it has an adequate, well-trained, and willing epidemiologists’ workforce.

## Roundtable Major Themes

The roundtable discussion provided an opportunity to explore the HSR concept; the importance of adapting to changing situations, including emergencies and crises; the tenets of a resilient health system; and the ways vulnerability can be reduced across and beyond health systems. It focused on how to create resilient and healthy communities and how to strengthen such resilience at the individual, community, and system levels. Finally, the region’s collective experience, the most important lessons learned, and how existing health systems can benefit from these lessons were discussed in particular.

The panel members highlighted the importance of enhancing HSR and provided their recommendations in the following 3 major areas: (1) global involvement, health systems, and governance; (2) research, innovation, and workforce capacity development; and (3) addressing inequities and engaging communities. The activities classified under these major areas are as follows:

- Global involvement, health systems, and governance:
  Leverage the current response to strengthen both pandemic preparedness and health systems.
  Invest in EPHFs, including those needed for all-hazards emergency risk management.
  Strengthen primary health care approaches for health security and universal health coverage.
  Improve organization and functioning of health systems for pandemic preparedness.
  Invest in addressing foundational health system gaps and essential public health functions for emergency management.
  Provide equal priority for maintaining essential health services and ensuring emergency preparedness and response.
  Increase domestic and global investment in health system foundations and all-hazards emergency risk management.
  Ensure good governance and leadership for effective emergency risk management with multisectoral coordination.
- Research, innovation, and workforce capacity development:
  Promote enabling environments for research, innovation, and learning.
  Use technology and new ways of organizing health services to provide alternative platforms for health service delivery and epidemic response.
  Adopt health workforce interventions to increase health systems resilience, such as increasing the number and quality of field epidemiologists in the public health workforce to ensure an adequate, well-trained, and willing epidemiologists’ workforce; task-shifting; and training of community health workers to assume basic public health functions.
- Addressing inequities and engaging communities:
  Address preexisting inequities and the disproportionate impact of COVID-19 on marginalized and vulnerable populations.
  Build and maintain public trust through community engagement and participation.
  Invest in institutionalized mechanisms for whole-of-society engagement.

## Conclusions

In conclusion, the lessons learned from COVID-19 response can reshape our thinking and strategies for approaching HSR. Considering the current situation in the EMR, there is a need to strengthen both pandemic preparedness and health systems, through investing in the essential public health functions, including those required for all-hazards emergency risk management. Institutionalized mechanisms for whole-of-society engagement strengthening the primary health care approaches for health security and universal health coverage, and promoting enabling environments for research, innovation, and learning should be ensured. Finally, investing in building epidemiological capacity through workforce development and continuous support to FETPs to work toward strengthening surveillance systems and participating in regional and global efforts in early response to outbreaks is needed.

The experience in the EMR and beyond has clearly shown the critical role of the health workforce in ensuring resilience. Capacity building for the public health workforce, in particular the FETP, proved to be instrumental in this respect. The implications for countries and the global community should include the development of a vision and strategy for enhancing resilience, streamlining and harmonizing global and country-level efforts, as well as mobilizing funding and resources for the effective implementation of the required interventions.

## Abbreviations

EMPHNET: Eastern Mediterranean Public Health Network
EMR: Eastern Mediterranean Region
FETP: Field Epidemiology Training Program
GHD: Global Health Development
HSR: health system resilience
NPHI: national public health institute
